# Differences in IGF-axis protein expression and survival among multiethnic breast cancer patients

**DOI:** 10.1002/cam4.375

**Published:** 2015-01-26

**Authors:** Brenda Y Hernandez, Lynne R Wilkens, Loïc Le Marchand, David Horio, Clayton D Chong, Lenora W M Loo

**Affiliations:** 1 University of Hawaii Cancer Center, University of HawaiiHonolulu, Hawaii; 2 John A. Burns School of Medicine, University of HawaiiHonolulu, Hawaii; 3 The Queen's Medical CenterHonolulu, Hawaii

**Keywords:** Breast cancer, IGF1, IGF1R, IGFBP2, IGFBP3, insulin-axis protein, multiethnic

## Abstract

There is limited knowledge about the biological basis of racial/ethnic disparities in breast cancer outcomes. Aberrations in IGF signaling induced by obesity and other factors may contribute to these disparities. This study examines the expression profiles of the insulin-like growth factor (IGF)-axis proteins and the association with breast cancer survival across a multiethnic population. We examined the expression profiles of the IGF1, IGF1R, IGFBP2 (IGF-binding proteins), and IGFBP3 proteins in breast tumor tissue and their relationships with all-cause and breast cancer-specific survival up to 17 years postdiagnosis in a multiethnic series of 358 patients in Hawaii, USA. Native Hawaiians, Caucasians, and Japanese were compared. Covariates included demographic and clinical factors and ER/PR/HER2 (estrogen receptor/progesterone receptor/human epidermal growth factor receptor-2) status. In Native Hawaiian patients, IGFBP2 and IGFBP3 expression were each independently associated with overall and breast cancer mortality (IGFB2: HR_mort_ = 10.96, 95% CI: 2.18–55.19 and HR_mort_ = 35.75, 95% CI: 3.64–350.95, respectively; IGFBP3: HR_mort_ = 5.16, 95% CI: 1.27–20.94 and HR_mort_ = 8.60, 95% CI: 1.84–40.15, respectively). IGF1R expression was also positively associated with all-cause mortality in Native Hawaiians. No association of IGF-axis protein expression and survival was observed in Japanese or Caucasian patients. The interaction of race/ethnicity and IGFBP3 expression on mortality risk was significant. IGF-axis proteins may have variable influence on breast cancer progression across different racial/ethnic groups. Expression of binding proteins and receptors in breast tumors may influence survival in breast cancer patients by inducing aberrations in IGF signaling and/or through IGF-independent mechanisms. Additional studies to evaluate the role of the IGF-axis in breast cancer are critical to improve targeted breast cancer treatment strategies.

## Introduction

Breast cancer is the most common cancer among women in the United States.^1^ The disease burden in terms of incidence and mortality, however, varies substantially across racial/ethnic populations.^1^ Poorer breast cancer survival is observed among premenopausal African-American women compared to Caucasian and Asian women.^2^–^4^ Substantial disparities are also observed in smaller minority populations in the United States, including Native Hawaiians who have amongst the highest breast cancer incidence and mortality rates in the nation.^5^ Racial/ethnic differences in the burden of breast cancer are not completely explained by established risk factors. Potential biologic mechanisms underlying these disparities have not been widely evaluated.

Obesity is a risk factor for the development of postmenopausal breast cancer,^6^,^7^ and there is evidence that obesity is associated with poorer breast cancer survival.^8^–^11^ Obesity disproportionately affects African-Americans and Native Hawaiians compared to other racial/ethnic groups in the United States.^12^–^14^ There is additional evidence that insulin resistance is associated with poor breast cancer outcome.^15^ Mechanisms related to obesity and subsequent aberrations in insulin signaling may contribute to the differences in breast cancer outcomes across racial/ethnic populations.

The insulin-like growth factor (IGF) signaling pathway has been associated with both initiation and progression of breast cancer.^16^ IGF1 and IGF2 signal through tyrosine kinase receptors, insulin receptors (IR) and IGF receptors 1 and 2 (IGF1R and IGF2R). The majority of biological effects of IGF signaling are mediated by IGF1R. For example, IGF1 acts as a mitogen in breast epithelial cells through its interaction with IGF1R. Bioavailability of the IGFs is modulated by six IGF-binding proteins (IGFBP). The primary IGFBP that binds to IGF1 is IGFBP3. Higher bioactive levels of IGF1, most accurately represented by the molecular ratio of circulating IGF1 to IGFBP3, have been shown to be associated with breast cancer mortality.^17^

In addition to regulating IGF bioavailability, IGFBPs also have IGF-independent functions which impact cellular growth, survival, and migration.^18^ Higher circulating levels of IGFBP2 have been associated with poorer survival with cancers of the colon, brain, ovary, and prostate.^19^,^20^ Elevated circulating levels of IGFBP2 have also been observed in individuals with breast cancer, and increased IGFBP2 expression in breast tumors has been shown to correlate with poor breast cancer prognosis.^21^–^23^ IGFBP3 expression has also been associated with high-grade tumors, recurrence, and poor prognosis in breast cancer patients.^21^,^24^,^25^

## Methods

We examined the expression profiles of four major proteins involved in the IGF signaling pathway, IGF1, IGF1R, IGFBP2, and IGFBP3, in breast tumor tissue and their relationships with survival in a multiethnic population of breast cancer patients. The study was approved by the University of Hawaii Committee on Human Studies. The study population consisted of 358 invasive breast cancer cases diagnosed in 1995 in Hawaii, USA. All cases had no prior history of breast cancer. Cases were part of a previously constructed tissue microarray (TMA) comprised of all formalin-fixed, paraffin-embedded (FFPE) tumor tissue specimens available from the Hawaii Residual Tissue Repository (RTR) of the National Cancer Institute's (NCI's) Surveillance, Epidemiology, and End-Results (SEER) program.^26^,^27^ The study population represents 51% of all female breast cancers diagnosed in the state in 1995 and is largely representative of cases statewide with respect to demographic and clinical characteristics.^27^ Specimens are annotated with deidentified, high-quality data from the Hawaii Tumor Registry of the NCI SEER program including demographic and clinical information and survival through 2012. Breast cancer TMA cases were previously assayed for expression of estrogen receptor (ER), progesterone receptor (PR), and human epidermal growth factor receptor-2 (HER-2)^27^ and data on these markers were generously made available for the present study by Dr. William Anderson of the National Cancer Institute.

**Figure 1:**
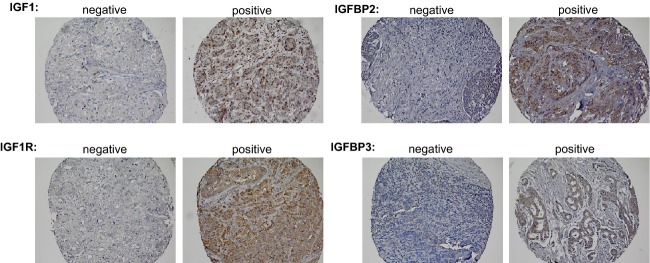
Immunohistochemical staining of IGF-axis proteins in breast cancer tissue. Negative and positive staining for IGF1, IGF1R, IGFBP2, and IGFBP3 expression. Individual tissue cores at 20× magnification.

Immunohistochemistry (IHC) utilized commercially available antibodies for IGF1 (polyclonal, dilution 1:1000; Abcam, Cambridge, MA), IGF1R*β* (polyclonal, dilution 1:50; Santa Cruz Biotechnology, Dallas, TX), IGFBP2 (polyclonal, dilution 1:25; Cell Signaling, Danvers, MA), and IGFBP3 (monoclonal, dilution 1:50; Calbiochem/Merck KGaA, Darmstadt, Germany) (Fig.1). IHC procedures were performed according to manufacturers’ protocols. IHC stains of human placental tissue was used as a positive control for IGF1R, IGFBP2, IGFBP3 staining, and human liver tissue for IGF1, IGF1R, IGFBP2, IGFBP3 staining. Breast tumor tissue with no primary antibody was used for negative control staining. On the TMA, each breast cancer case was represented by up to four 0.6 mm cores of tumor tissue. Slides were evaluated by one pathologist (D. H.) based on the intensity of cytoplasmic staining and the percentage of cells stained. Each core was scored as positive, weakly positive/equivocal, and negative. Cases were considered positive based on positive staining of at least one core. Cases with insufficient tissue or inadequate IHC results were excluded from the statistical analyses. In addition, cases with equivocal IHC results were excluded from analyses specific to that protein.

Comparisons between categorical variables utilized the Pearson chi-square test. Evaluation stratified by race/ethnicity included the three largest groups (Caucasian, Japanese, Native Hawaiian). Survival time was defined from the date of diagnosis to the date of last follow-up or death. Cases who were alive as of 2012 or were lost to follow-up were censored at the date of last follow-up. Overall survival was evaluated based on all causes of death. Breast cancer-specific survival was evaluated based on death from breast cancer; subjects who died of causes other than breast cancer were censored at time of death. Kaplan–Meier curves and log-rank tests were used to compare survival distribution by protein expression, without adjustment and with total follow-up of 15 years. Risk of mortality, measured as hazard ratios (HR) and 95% confidence intervals (CI), adjusted for potential confounders, was calculated via Cox proportional hazards regression. Cases negative for protein expression were set as the reference in examining the association of the protein markers with survival. Potential confounders, included as covariates in the log-linear model, were age (<50, ≥50 years), stage (localized, regional involvement/distant metastasis), first course of treatment (surgery only, surgery plus other treatment and/or other treatment), and receptor status: ER (+/−), PR (+/−), and HER2 (+/−). Race/ethnicity was included as an additional covariate in a separate model (represented as indicator variables with Caucasians as the reference). Interaction was tested by the Wald test of cross-product terms of race/ethnicity and IGF-axis proteins entered into the models.

## Results

### Study population characteristics

The 358 breast cancer cases were primarily 50 years and older (77%) and were comprised of Japanese (34%), Caucasians (29%), Native Hawaiians (16%), and other race/ethnic groups (21%) (Table1). Tumors were predominantly infiltrating ductal carcinomas (84%), of localized stage (68%), and of moderate or poor differentiation (73%). The majority of tumors expressed ER (72%) and PR (62%); 19% expressed HER2. Surgery combined with radiation with or without other therapy comprised the first course of treatment for 54% of cases. Fifty-one percent of cases were alive 17 years post-diagnosis. Of the 177 deaths, 66 were due to breast cancer. Age, stage, histology, grade, treatment, and receptor status (ER, PR, and HER2) did not vary across race/ethnic groups (data not shown).

**Table 1:** Characteristics of invasive breast cancer cases

	All (*n* = 358)	
	No.	Percent of total^1^
Age group		
25–49	83	23.2
50–69	184	51.4
≥70	91	25.4
Race/ethnicity		
Caucasian	105	29.3
Japanese	122	34.1
Native Hawaiian	56	15.6
Other^2^	75	21.0
Stage^3^		
Localized	243	67.9
Advanced regional involvement	105	29.3
Distant metastases	10	2.8
Histology		
Infiltrating ductal carcinoma^4^	302	84.4
Lobular carcinoma	17	4.8
Mucinous adenocarcinoma	11	3.1
Other	28	7.8
Grade		
Well-differentiated	34	9.5
Moderately differentiated	130	36.3
Poorly-/undifferentiated	131	36.6
Unknown	63	17.6
ER^5^		
Negative	90	27.7
Positive	235	72.3
PR^5^		
Negative	123	37.8
Positive	202	62.2
HER2^5^		
Negative	265	80.8
Positive	63	19.2
ER, PR and HER2^5^		
ER−/PR−/HER2−	48	14.7
ER+ and/or PR+ and/or HER2+	279	85.3
First course of treatment		
Surgery only	64	17.9
Surgery and radiation (with or without other therapy)	193	53.9
Surgery and other treatment	97	27.1
Other	4	1.1
Vital status^6^		
Alive	181	50.6
Deceased- breast cancer	66	18.4
Deceased- other causes	111	31.0

ER, estrogen receptor; PR, progesterone receptor; HER-2, human epidermal receptor-2.

1 Total percent may be slightly lower or higher than 100 due to rounding.

2 Includes Chinese, Filipina, Other Asian, Other Pacific Islander, and other race/ethnic groups.

3 Based on SEER extent of disease; advanced disease includes regional involvement and distant metastases.

4 Includes nine cases of infiltrating ductal carcinoma plus other histologic types.

5 Excludes cases with inadequate IHC results.

6 After 17 years of follow-up post-diagnosis.

### IGF-axis protein expression and patient and clinical characteristics

IGF1 was expressed in 30% of breast tumors, IGF1R in 26%, IGFBP2 in 74%, and IGFBP3 in 32% (Table2). The number of specimens excluded from the analysis because of missing results due to insufficient tissue, inadequate IHC results or equivocal IHC results are given in a footnote to the table. IGF1R was positively associated with expression of IGFBP2 (*P* < 0.0001) and IGFBP3 (*P* < 0.0001). Expression of IGFBP2 and IGFBP3 were positively associated (*P* < 0.0001). IGF1 expression was not associated with expression of IGF1R (*P* = 0.46), IGFBP2 (*P* = 0.15), or IGFBP3 (*P* = 0.47).

**Table 2:** IGF-axis protein expression in breast tumors by patient and clinical characteristics

	Total^2^	IGF1^1^		IGF1R^1^		IGFBP2^1^		IGFBP3^1^	
		Positive	*P*-value^3^	Positive	*P*-value^3^	Positive	*P*-value^3^	Positive	*P*-value^3^
All cases	358	30%		26%		74%		32%	
Age									
<50	83	30%	0.93	30%	0.93	75%	0.90	35%	0.60
≥50	275	29%		27%		74%		32%	
Race/ethnicity^4^									
Caucasian	105	27%	0.10	25%	0.08	73%	0.73	29%	0.39
Japanese	122	34%		37%		76%		39%	
Native Hawaiian	56	16%		20%		79%		34%	
Stage									
Localized	243	32%	0.24	28%	0.55	72%	0.29	32%	0.85
Regional/distant	115	25%		24%		78%		32%	
Histology									
Infiltrating ductal carcinoma	302	30%	0.97	28%	0.08	76%	0.18	33%	0.53
Other	56	29%		16%		66%		28%	
Grade									
Well-/moderately differentiated	164	33%	**0.009**	26%	0.44	74%	0.61	33%	0.47
Poorly-/undifferentiated	194	17%		30%		77%		37%	
ER/PR/HER2^5^									
ER−	90	19%	**0.02**	15%	**0.01**	58%	**0.0002**	22%	**0.02**
ER+	235	35%		32%		81%		37%	
PR−	123	21%	**0.01**	13%	**<0.0001**	62%	**0.001**	21%	**0.002**
PR+	202	37%		36%		82%		40%	
HER2−	265	29%	0.60	26%	0.84	70%	**0.002**	27%	**0.001**
HER2+	63	33%		28%		91%		52%	
ER−/PR−/HER2−	48	8%	**0.002**	18%	0.16	46%	**<0.0001**	18%	**0.03**
ER+ and/or PR+ and/or HER2+	279	34%		28%		79%		35%	

IGF, insulin-like growth factor; ER, estrogen receptor; PR, progesterone receptor; HER-2, human epidermal receptor-2; IGFBP, insulin-like growth factor-binding proteins; IHC, Immunohistochemistry.

1 Excludes cases with insufficient tissue or inadequate or equivocal IHC results (*n* = 111, IGF1; *n* = 74, IGF1R; *n* = 77, IGFBP2; *n* = 88, IGFBP3).

2 Row category total.

3 Statistically significant values (*P* < 0.05) shown in bold.

4 Excludes Chinese, Filipina, other Asian, other Pacific Islander, and other race/ethnic groups.

5 Excludes cases with inadequate IHC results.

The expression of IGF-axis proteins did not significantly vary by age, race/ethnicity, stage, or histology (Table2). IGF1 expression varied by tumor grade: IGF1 positivity was higher in well-differentiated and moderately differentiated tumors (33%) compared to poorly differentiated and undifferentiated tumors (17%) (*P* = 0.009). IGF-axis protein expression varied with ER, PR, and HER2 status. Each of the four proteins were more highly expressed in ER-positive compared to ER-negative tumors (*P* = 0.0002–0.02) and in PR-positive compared to PR-negative tumors (*P* < 0.0001–0.01). IGFBP2 and IGFBP3 were each more highly expressed in HER2-positive relative to HER2-negative tumors (*P* = 0.002 and 0.001, respectively). Compared to tumors positive for at least one hormone receptor among ER, PR, and HER2, triple negative tumors (ER-/PR-/HER2-) were significantly less likely to express IGF1 (*P* = 0.002), IGFBP2 (*P* < 0.0001), and IGFBP3 (*P* = 0.03).

### IGF-axis protein expression and survival

**Figure 2:**
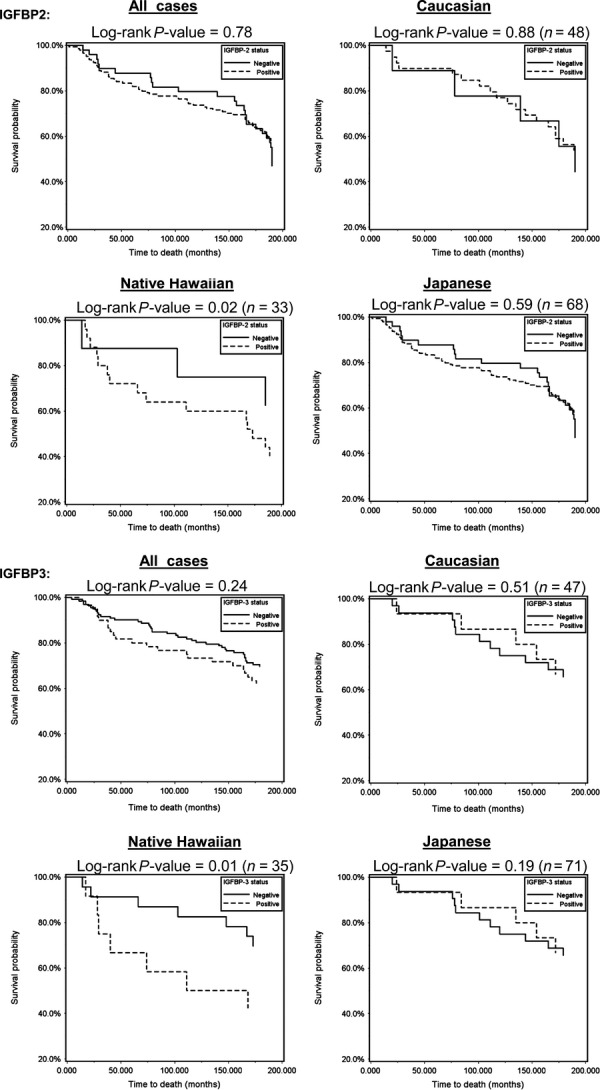
Breast tumor tissue expression of IGFBP2 and IGFBP3 and breast cancer-specific survival. Kaplan–Meier curves and log-rank tests were used to compare survival distribution by protein expression of IGFBP2 and IGFBP3, without adjustment and up to 180 months of follow-up.

Across all cases, overall survival and breast cancer-specific survival, unadjusted for confounders, did not vary by expression of IGF1, IGF1R, IGFBP2, or IGFBP3 (Fig.2). Similarly, survival differences by IGF protein expression were also not observed when examined separately in women <50 and ≥50 years old (data not shown). When examined separately by individual race/ethnic groups, survival did not vary by individual protein expression for Caucasians or Japanese. However, among Native Hawaiians, survival differences were observed for IGFBP2 and IGFBP3 expression. Breast cancer-specific survival was shorter for Native Hawaiians with IGFBP2-positive compared to those with IGFPB2-negative tumors (Log-rank *P* = 0.02) (Fig.2A). Native Hawaiians with IGFBP3-positive tumors had poorer overall and breast cancer-specific survival compared to IGFBP3-negative tumors (Log-rank *P* = 0.03 and Log-rank *P* = 0.01, respectively) (Fig.2B).

The relationship of IGF-axis protein expression with mortality was examined adjusting for age, stage, treatment, ER, PR, and HER2 (and race/ethnicity in a separate model) (Table3). In Native Hawaiians, IGF1R expression was positively associated with risk of all-cause death (HR_mort_ = 7.42, 95% CI 1.36–40.41). IGFBP2 expression was associated with overall and breast cancer mortality (HR_mort_ = 10.96, 95% CI: 2.18–55.19 and HR_mort_ = 35.75, 95% CI: 3.64–350.95, respectively). Similarly, cases expressing IGFBP3 were at higher risk of all-cause and breast cancer mortality (HR_mort_ = 5.16, 95% CI: 1.27–20.94 and HR_mort_ = 8.60, 95% CI: 1.84–40.15), respectively). No association of IGF-axis protein expression and risk of mortality was observed in Caucasian or Japanese women.

**Table 3:** IGF-axis protein expression and risk of mortality in breast cancer cases by race/ethnicity

Protein^1^	Survival	All (*n* = 358)		All (*n* = 358)		Caucasian (*n* = 105)		Native Hawaiian (*n* = 56)		Japanese (*n* = 122)		*P*-value for Interaction^5^
		Adjusted^1^		Adjusted[Table-fn tf3-3]		Adjusted^1^		Adjusted^1^		Adjusted^1^		
		HR^4^	95% CI	HR^4^	95% CI	HR^4^	95% CI	HR^4^	95% CI	HR^4^	95% CI	
IGF1+	Overall	0.91	0.61–1.38	0.99	0.58–1.34	0.7	0.26–1.90	1.13	0.22–5.75	0.77	0.36–1.63	0.26
	Breast cancer	1.09	0.72–1.65	1.04	0.68–1.59	0.7	0.26–1.88	1.34	0.31–5.77	1.21	0.56–2.64	0.45
IGF1R+	Overall	1.04	0.67–1.60	1.13	0.72–1.78	1.14	0.45–2.88	**7.42**	**1.36**–**40.41**	0.67	0.33–1.35	0.43
	Breast cancer	1.2	0.78–1.85	1.30	0.83–2.03	1.13	0.45–2.84	5.41	0.87–33.55	0.93	0.46–1.87	0.72
IGFBP2+	Overall	0.83	0.54–1.28	0.76	0.49–1.19	0.77	0.27–2.17	**10.96**	**2.18**–**55.19**	0.82	0.40–1.70	0.49
	Breast cancer	0.92	0.60–1.42	0.85	0.54–1.33	0.76	0.27–2.13	**35.75**	**3.64**–**350.95**	0.97	0.46–2.04	1.32
IGFBP3+	Overall	1.18	0.79–1.77	1.24	0.82–1.87	0.56	0.22–1.40	**5.16**	**1.27**–**20.94**	1.35	0.67–2.70	0.04
	Breast cancer	1.17	0.78–1.77	1.24	0.82–1.87	0.56	0.22–1.41	**8.6**	**1.84–40.15**	1.37	0.69–2.75	**0.03**

IGF, insulin-like growth factor; ER, estrogen receptor; PR, progesterone receptor; HER-2, human epidermal receptor-2; IGFBP, IGF-binding proteins; HR, hazard ratios; IHC, Immunohistochemistry.

1 Models adjusted for age, stage, first course treatment, ER, PR, HER2.

2 Models adjusted for age, stage, first course treatment, ER, PR, HER2, and race/ethnicity (indicator variables with white as reference).

3 Excludes cases with insufficient tissue or inadequate or equivocal IHC results (*n* = 111, IGF1; *n* = 74, IGF1R; *n* = 77, IGFBP2; *n* = 88, IGFBP3).

4 Hazard ratio (HR); reference includes cases negative for protein(s) of interest; statistically significant values (*P* < 0.05) shown in bold.

5 Based on Wald test of cross-product terms of race/ethnicity and protein expression; statistically significant values (*P *< 0.05) shown in bold.

For all-cause and breast cancer-specific mortality, no heterogeneity of effects was observed by race/ethnicity for IGF1 (*P* for interaction 0.26 and 0.45, respectively), IGF1R (*P* for interaction 0.43 and 0.72), or IGFBP2 (*P* for interaction 0.49 and 0.32). There was evidence of interaction between race/ethnicity and IGFBP3 (*P* for interaction 0.04 and 0.03, respectively).

## Discussion

This is the first study to evaluate the relationship of tissue expression of IGF1, IGFR1, IGFBP2, and IGFBP3 and survival in US breast cancer patients of Asian, Pacific Islander, and Caucasian ancestry. IGF-axis protein expression was not associated with mortality in Japanese and Caucasian patients. IGF-axis protein expression was a predictor of mortality risk in Native Hawaiian breast cancer patients—a group who suffer disparately high incidence and mortality rates for this cancer.^5^ In Native Hawaiian patients, IGFBP2 and IGFBP3 were each independently associated with all-cause and breast cancer-specific mortality, and IGF1R was associated with death from all causes.

Previous studies, primarily focused on circulating levels, have found that IGFBP2 and IGFBP3 are associated with poor survival and poor prognostic characteristics in breast cancer.^21^–^25^ IGFBP2 and IGFBP3 regulate the availability of free IGF1. Png et al. demonstrated that IGFBP2 recruits endothelial cells to metastatic breast cancer cells by modulating IGF1-mediated activation of IGF1R.^28^ As the primary receptor for IGF1, IGF1R is a key regulator of IGF signaling, including the mitogenic effects of IGF1 in breast tissue.^16^

Our results indicate that IGF-axis protein profiles in breast tumor tissue may have differential effects on survival in patients of diverse racial and ethnic backgrounds. The basis of this difference can only be speculated. Based on previous reports there are dramatic differences in the prevalence of obesity in these populations. Among healthy adults, the highest prevalence is in Native Hawaiian women (28%), followed by Caucasians (12%), and Japanese (4%).^12^ A greater proportion of Native Hawaiian breast cancer patients have a history of obesity (40%) compared to Caucasian, Japanese, and Latino breast cancer patients (*P* < 0.001).^8^

Obesity is associated with poor survival in breast cancer 8–11 and Native Hawaiian breast cancer patients have among the poorest survival in the United States.^5^ Our results suggest that IGF-axis proteins may have variable influence on breast cancer progression depending on the level of obesity in populations. It is possible that the very high prevalence of obesity among Native Hawaiian patients result in the disruption of IGF-axis signaling that contributes to poorer survival.

Consistent with our findings in Native Hawaiians, Probst-Hensch et al. observed an association of overweight with overall survival only in breast cancer patients with IGFBP2-positive tumors and found that IGFBP3 was correlated with BMI.^21^ It is possible that women with preexisting obesity are more likely to express IGFBP2. Our results are consistent with an important role played by insulin-axis proteins in the pathogenesis of breast cancer. Moreover, adiposity may be a key mediator of this relationship.

The poor outcome in Native Hawaiian breast cancer patients does not appear to be attributed to ER/PR status as they do not have a preponderance of more aggressive ER/PR-negative tumors.^29^ All four of the IGF-axis proteins were expressed more frequently in ER-positive tumors. Our results were consistent with previous observations that IGFBP2 is highly expressed in ER-positive and rarely in ER-negative tumors.^21^

In a recent report, Foulstone et al. observed a positive feedback loop between IGFBP2 and ER-*α*, where stimulation of ER-*α* increased IGFBP2 production and in turn IGFBP2 production influences the expression of ER-*α*.^30^ IGFBP2 is believed to mediate ER-*α* expression in an IGF-independent function through interactions with integrins. IGFBP2 interacts with the *α*5*β*1-integrin receptor through an Arg-Gly-Asp (RGD) peptide sequence at its C-terminus 31 and the IGFBP2-integrin interaction has been observed to lead to reduction in the tumor suppressor phosphatase and tensin homolog (PTEN).^32^ In addition, ER-positive breast cancers resistant to hormone therapy, such as Tamoxifen, overexpress IGFBP2.^33^,^34^

Delineating IGFBP3's prognostic role in breast cancer survival is complicated due to its ability to both promote and inhibit cellular proliferation. IGFBP3 levels are modulated by TGF*β* and both proteins act as inhibitors of proliferation.^35^ IGFBP3 can also interact with the epidermal growth factor receptor (EGFR),^36^ integrins,^37^ and caveolin-1^38^ to promote proliferation. IGFBP3 was also recently shown to play a role in a key mechanism which makes tumors resistant to DNA-damaging agents such as etoposides and doxorubicin.^39^

Our study was unique in a number of respects. The study population included Asians and Pacific Islanders not widely represented in US breast cancer studies. Breast cancer patients were diagnosed in the same calendar year within one state. This minimized the potential influence of temporal and geographic variation in breast cancer diagnosis and treatment. Finally, with vital status data up to 17 years postdiagnosis, we were able to assess long-term survivorship.

There were a number of study limitations. The small numbers of cases limited ethnic-specific comparisons. As SEER registry data are confined to first course of treatment, we were unable to consider the complete treatment history in the survival analyses. Protein expression was broadly defined as positive or negative, without more refined quantification of expression level. It is possible that high-level protein expression were most strongly associated with breast cancer outcome. A major limitation was the lack of patient information on body size that would permit evaluation of the relationship of insulin-axis protein expression by obesity status.

Our findings suggest that there are racial/ethnic differences in the bioavailability of IGFs through the variable expression of binding proteins and receptors in breast tumors. These differences may manifest as aberrations in IGF signaling which can negatively influence survival in breast cancer patients. Racial/ethnic differences in the expression of binding proteins and receptors in breast tumors may be influenced by variation in obesity and other risk factors. Additional studies to evaluate the role of the IGF-axis and obesity in breast cancer are critical to increase our understanding and to improve treatment strategies.
